# Guessing with Distributed Encoders

**DOI:** 10.3390/e21030298

**Published:** 2019-03-19

**Authors:** Annina Bracher, Amos Lapidoth, Christoph Pfister

**Affiliations:** 1 P&C Solutions, Swiss Re, 8022 Zurich, Switzerland; 2 Signal and Information Processing Laboratory, ETH Zurich, 8092 Zurich, Switzerland

**Keywords:** Arimoto–Rényi conditional entropy, distributed source coding, guessing, Rényi entropy

## Abstract

Two correlated sources emit a pair of sequences, each of which is observed by a different encoder. Each encoder produces a rate-limited description of the sequence it observes, and the two descriptions are presented to a guessing device that repeatedly produces sequence pairs until correct. The number of guesses until correct is random, and it is required that it have a moment (of some prespecified order) that tends to one as the length of the sequences tends to infinity. The description rate pairs that allow this are characterized in terms of the Rényi entropy and the Arimoto–Rényi conditional entropy of the joint law of the sources. This solves the guessing analog of the Slepian–Wolf distributed source-coding problem. The achievability is based on random binning, which is analyzed using a technique by Rosenthal.

## 1. Introduction

In the Massey–Arıkan guessing problem [1,2], a random variable *X* is drawn from a finite set X according to some probability mass function (PMF) $P_{X}$, and it has to be determined by making guesses of the form “Is *X* equal to *x*?” until the guess is correct. The guessing order is determined by a guessing function *G*, which is a bijective function from X to {1,…,|X|}. Guessing according to *G* proceeds as follows: the first guess is the element ${\hat{x}}_{1}\in X$ satisfying $G({\hat{x}}_{1})=1$; the second guess is the element ${\hat{x}}_{2}\in X$ satisfying $G({\hat{x}}_{2})=2$, and so on. Consequently, G(X) is the number of guesses needed to guess *X*. Arıkan [2] showed that for any ρ>0, the ρth moment of the number of guesses required by an optimal guesser $G^{*}$ to guess *X* is bounded by:

(1) $$\begin{array}{c} \frac{1}{{\left(1+\ln\;\left|X\right|\right)}^{\rho }}\;2^{\rho H_{1/(1+\rho )}\left(X\right)}\leq E\left[G^{*}{\left(X\right)}^{\rho }\right]\leq 2^{\rho H_{1/(1+\rho )}\left(X\right)}, \end{array}$$

where ln(·) denotes the natural logarithm, and $H_{1/(1+\rho )}\left(X\right)$ denotes the Rényi entropy of order $\frac{1}{1+\rho }$, which is defined in Section 3 ahead (refinements of (1) were recently derived in [3]).

**Figure 1:**
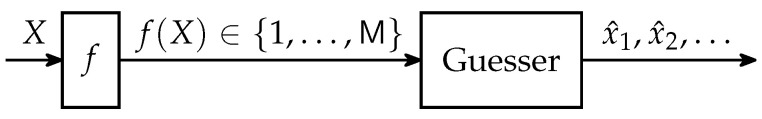
Guessing with an encoder *f*.

Guessing with an encoder is depicted in Figure 1. Here, prior to guessing *X*, the guesser is provided some side information about *X* in the form of f(X), where f:X→{1,…,M} is a function taking on at most M different values (“labels”). Accordingly, a guessing function G(·|·) is a function from X×{1,…,M} to {1,…,|X|} such that for every label m∈{1,…,M}, G(·|m):X→{1,…,|X|} is bijective. If, among all encoders, $f^{*}$ minimizes the ρth moment of the number of guesses required by an optimal guesser to guess *X* after observing f(X), then [4] (Corollary 7):

(2) $$\begin{array}{c} \frac{1}{{\left(1+\ln\;\left|X\right|\right)}^{\rho }}\;2^{\rho [H_{1/(1+\rho )}\left(X\right)-\log M]}\leq E\left[G^{*}{\left(X|f^{*}\left(X\right)\right)}^{\rho }\right]\leq 1+2^{\rho [H_{1/(1+\rho )}\left(X\right)-\log M+1]}. \end{array}$$

Thus, in guessing a sequence of independent and identically distributed (IID) random variables, a description rate of approximately $H_{1/(1+\rho )}\left(X\right)$ bits per symbol is needed to drive the ρth moment of the number of guesses to one as the sequence length tends to infinity [4,5] (see Section 2 for more related work).

**Figure 2:**
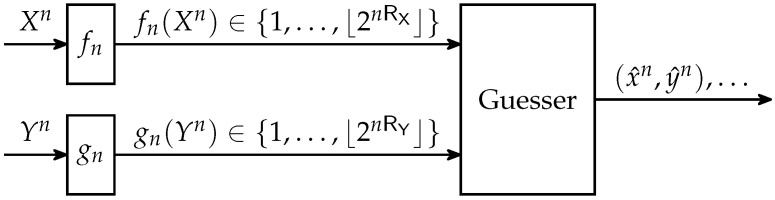
Guessing with distributed encoders $f_{n}$ and $g_{n}$.

In this paper, we generalize the single-encoder setting from Figure 1 to the setting with distributed encoders depicted in Figure 2, which is the analog of Slepian–Wolf coding [6] for guessing: A source generates a sequence of pairs ${\left\{\left(X_{i},Y_{i}\right)\right\}}_{i=1}^{n}$ over a finite alphabet X×Y. The sequence $X^{n}$ is described by one of $\left\lfloor 2^{nR_{X}}\right\rfloor$ labels and the sequence $Y^{n}$ by one of $\left\lfloor 2^{nR_{Y}}\right\rfloor$ labels using functions:

(3) $$\begin{array}{cc} f_{n}:X^{n} & \to \{1,\ldots ,\left\lfloor 2^{nR_{X}}\right\rfloor \}, \end{array}$$

(4) $$\begin{array}{cc} g_{n}:Y^{n} & \to \{1,\ldots ,\left\lfloor 2^{nR_{Y}}\right\rfloor \}, \end{array}$$

where $R_{X}\geq 0$ and $R_{Y}\geq 0$. Based on $f_{n}\left(X^{n}\right)$ and $g_{n}\left(Y^{n}\right)$, a guesser repeatedly produces guesses of the form $\left({\hat{x}}^{n},{\hat{y}}^{n}\right)$ until $\left({\hat{x}}^{n},{\hat{y}}^{n}\right)=\left(X^{n},Y^{n}\right)$.

For a fixed ρ>0, a rate pair $\left(R_{X},R_{Y}\right)\in R_{\geq 0}^{2}$ is called achievable if there exists a sequence of encoders and guessing functions ${\left\{\left(f_{n},g_{n},G_{n}\right)\right\}}_{n=1}^{\infty }$ such that the ρth moment of the number of guesses tends to one as *n* tends to infinity, i.e.,

(5) $$\begin{array}{c} \lim_{n\to \infty }E\left[G_{n}{\left(X^{n},Y^{n}|f_{n}\left(X^{n}\right),g_{n}\left(Y^{n}\right)\right)}^{\rho }\right]=1. \end{array}$$

Our main contribution is Theorem 1, which characterizes the achievable rate pairs. For a fixed ρ>0, let the region R(ρ) comprise all rate pairs $\left(R_{X},R_{Y}\right)\in R_{\geq 0}^{2}$ satisfying the following inequalities simultaneously:

(6) $$\begin{array}{cc} R_{X} & \geq \underset{n\to \infty }{lim sup}\frac{H_{\tilde{\rho }}\left(X^{n}|Y^{n}\right)}{n}, \end{array}$$

(7) $$\begin{array}{cc} R_{Y} & \geq \underset{n\to \infty }{lim sup}\frac{H_{\tilde{\rho }}\left(Y^{n}|X^{n}\right)}{n}, \end{array}$$

(8) $$\begin{array}{cc} R_{X}+R_{Y} & \geq \underset{n\to \infty }{lim sup}\frac{H_{\tilde{\rho }}\left(X^{n},Y^{n}\right)}{n}, \end{array}$$

where the Rényi entropy $H_{\alpha }\left(\cdot \right)$ and the Arimoto–Rényi conditional entropy $H_{\alpha }\left(\cdot |\cdot \right)$ of order α are both defined in Section 3 ahead, and throughout the paper,

(9) $$\begin{array}{c} \tilde{\rho }≜\frac{1}{1+\rho }. \end{array}$$

**Theorem** **1.** *For any ρ>0, all rate pairs in the interior of R(ρ) are achievable, while those outside R(ρ) are not. If ${\left\{\left(X_{i},Y_{i}\right)\right\}}_{i=1}^{\infty }$ are IID according to $P_{XY}$, then *(6)*–*(8)* reduce to:*

(10) $$\begin{array}{cc} R_{X} & \geq H_{\tilde{\rho }}\left(X|Y\right), \end{array}$$

(11) $$\begin{array}{cc} R_{Y} & \geq H_{\tilde{\rho }}\left(Y|X\right), \end{array}$$

(12) $$\begin{array}{cc} R_{X}+R_{Y} & \geq H_{\tilde{\rho }}\left(X,Y\right). \end{array}$$

**Proof.**  The converse follows from Corollary 1 in Section 4; the achievability follows from Corollary 2 in Section 5; and the reduction of (6)–(8) to (10)–(12) in the IID case follows from (19) and (20) ahead. ☐

The rate region defined by (10)–(12) resembles the rate region of Slepian–Wolf coding [6] (Theorem 15.4.1); the difference is that the Shannon entropy and conditional entropy are replaced by their Rényi counterparts. The rate regions are related as follows:

**Remark** **1.** *For memoryless sources and ρ>0, the region R(ρ) is contained in the Slepian–Wolf region. Typically, the containment is strict.*

**Proof.**  The containment follows from the monotonicity of the Arimoto–Rényi conditional entropy: (9) implies that $\tilde{\rho }\in \left(0,1\right)$, so, by [7] (Proposition 5), $H_{\tilde{\rho }}\left(X|Y\right)\geq H\left(X|Y\right)$, $H_{\tilde{\rho }}\left(Y|X\right)\geq H\left(Y|X\right)$, and $H_{\tilde{\rho }}\left(X,Y\right)\geq H\left(X,Y\right)$. As for the strict containment, first note that the Slepian–Wolf region contains at least one rate pair $\left(R_{X},R_{Y}\right)$ satisfying $R_{X}+R_{Y}=H\left(X,Y\right)$. Consequently, if $H_{\tilde{\rho }}\left(X,Y\right)>H\left(X,Y\right)$, then the containment is strict. Because $H_{\tilde{\rho }}\left(X,Y\right)>H\left(X,Y\right)$ unless (X,Y) is distributed uniformly over its support [8], the containment is typically strict.

The claim can also be shown operationally: The probability of error is equal to the probability that more than one guess is needed, and for every ρ>0,

(13) $$\begin{array}{cc} \mathrm{Pr}\left[G_{n}\left(X^{n},Y^{n}|f_{n}\left(X^{n}\right),g_{n}\left(Y^{n}\right)\right)\geq 2\right] & =\mathrm{Pr}\left[G_{n}{\left(X^{n},Y^{n}|f_{n}\left(X^{n}\right),g_{n}\left(Y^{n}\right)\right)}^{\rho }-1\geq 2^{\rho }-1\right] \end{array}$$

(14) $$\begin{array}{c} \leq \frac{E\left[G_{n}{\left(X^{n},Y^{n}|f_{n}\left(X^{n}\right),g_{n}\left(Y^{n}\right)\right)}^{\rho }\;\right]-1}{2^{\rho }-1}, \end{array}$$

where (14) follows from Markov’s inequality. Thus, the probability of error tends to zero if the ρth moment of the number of guesses tends to one.  ☐

Despite the resemblance between (10)–(12) and the Slepian–Wolf region, there is an important difference: while Slepian–Wolf coding allows separate encoding with the same sum rate as with joint encoding, this is not necessarily true in our setting:

**Remark** **2.** *Although the sum rate constraint *(12)* is the same as in single-source guessing [5], separate encoding of $X^{n}$ and $Y^{n}$ may require a larger sum rate than joint encoding of $X^{n}$ and $Y^{n}$.*

**Proof.**  If $H_{\tilde{\rho }}\left(X|Y\right)+H_{\tilde{\rho }}\left(Y|X\right)>H_{\tilde{\rho }}\left(X,Y\right)$, then (10) and (11) together impose a stronger constraint on the sum rate than (12). For example, if:

$$\begin{array}{ccc} P_{XY}\left(x,y\right) & y=0 & y=1\\ x=0 & 0.65 & 0.17\\ x=1 & 0.17 & 0.01 \end{array}$$

and ρ=1, then $H_{1/2}\left(X|Y\right)+H_{1/2}\left(Y|X\right)\approx 1.61$ bits, so separate (distributed) encoding requires a sum rate exceeding 1.61 bits as opposed to joint encoding, which is possible with $H_{1/2}\left(X,Y\right)\approx 1.58$ bits (in Slepian–Wolf coding, this cannot happen because H(X,Y)−H(X|Y)−H(Y|X)=I(X;Y)≥0).  ☐

The guessing problem is related to the task-encoding problem, where based on $f_{n}\left(X^{n}\right)$ and $g_{n}\left(Y^{n}\right)$, the decoder outputs a list that is guaranteed to contain $\left(X^{n},Y^{n}\right)$, and the ρth moment of the list size is required to tend to one as *n* tends to infinity. While, in the single-source setting, the guessing problem and the task-encoding problem have the same asymptotics [4], this is not the case in the distributed setting:

**Remark** **3.** *For memoryless sources, the task-encoding region from [9] is strictly smaller than the guessing region R(ρ) unless X and Y are independent.*

**Proof.**  In the IID case, the task-encoding region is the set of all rate pairs $\left(R_{X},R_{Y}\right)\in R_{\geq 0}^{2}$ satisfying the following inequalities [9] (Theorem 1):

(15) $$\begin{array}{cc} R_{X} & \geq H_{\tilde{\rho }}\left(X\right), \end{array}$$

(16) $$\begin{array}{cc} R_{Y} & \geq H_{\tilde{\rho }}\left(Y\right), \end{array}$$

(17) $$\begin{array}{cc} R_{X}+R_{Y} & \geq H_{\tilde{\rho }}\left(X,Y\right)+K_{\tilde{\rho }}\left(X;Y\right), \end{array}$$

where $K_{\alpha }\left(X;Y\right)$ is a Rényi measure of dependence studied in [10] (when α is one, $K_{\alpha }\left(X;Y\right)$ is the mutual information). The claim now follows from the following observations: By [7] (Theorem 2), $H_{\tilde{\rho }}\left(X\right)\geq H_{\tilde{\rho }}\left(X|Y\right)$ with equality if and only if *X* and *Y* are independent; similarly, $H_{\tilde{\rho }}\left(Y\right)\geq H_{\tilde{\rho }}\left(Y|X\right)$ with equality if and only if *X* and *Y* are independent; and by [10] (Theorem 2), $K_{\tilde{\rho }}\left(X;Y\right)\geq 0$ with equality if and only if *X* and *Y* are independent.  ☐

The rest of this paper is structured as follows: in Section 2, we review other guessing settings; in Section 3, we recall the Rényi information measures and prove some auxiliary lemmas; in Section 4, we prove the converse theorem; and in Section 5, we prove the achievability theorem, which is based on random binning and, in the case ρ>1, is analyzed using a technique by Rosenthal [11].

## 2. Related Work

Tighter versions of (1) can be found in [3,12]. The large deviation behavior of guessing was studied in [13,14]. The relation between guessing and variable-length lossless source coding was explored in [3,15,16].

Mismatched guessing, where the assumed distribution of *X* does not match its actual distribution, was studied in [17], along with guessing under source uncertainty, where the PMF of *X* belongs to some known set, and a guesser was sought with good worst-case performance over that set. Guessing subject to distortion, where instead of guessing *X*, it suffices to guess an $\hat{X}$ that is close to *X* according to some distortion measure, was treated in [18].

If the guesser observes some side information *Y*, then the ρth moment of the number of guesses required by an optimal guesser is bounded by [2]:

(18) $$\begin{array}{c} \frac{1}{{\left(1+\ln\;\left|X\right|\right)}^{\rho }}\;2^{\rho H_{\tilde{\rho }}\left(X|Y\right)}\leq E\left[G^{*}{\left(X|Y\right)}^{\rho }\right]\leq 2^{\rho H_{\tilde{\rho }}\left(X|Y\right)}, \end{array}$$

where $H_{\tilde{\rho }}\left(X|Y\right)$ denotes the Arimoto–Rényi conditional entropy of order $\tilde{\rho }=\frac{1}{1+\rho }$, which is defined in Section 3 ahead (refinements of (18) were recently derived in [3]). Guessing is related to the cutoff rate of a discrete memoryless channel, which is the supremum over all rates for which the ρth moment of the number of guesses needed by the decoder to guess the message can be driven to one as the block length tends to infinity. In [2,19], the cutoff rate was expressed in terms of Gallager’s $E_{0}$ function [20]. Joint source-channel guessing was considered in [21].

Guessing with an encoder, i.e., the situation where the side information can be chosen, was studied in [4], where it was also shown that guessing and task encoding [22] have the same asymptotics. With distributed encoders, however, task encoding [9] and guessing no longer have the same asymptotics; see Remark 3. Lower and upper bounds for guessing with a helper, i.e., an encoder that does not observe *X*, but has access to a random variable that is correlated with *X*, can be found in [5].

## 3. Preliminaries

Throughout the paper, log(·) denotes the base-two logarithm. When clear from the context, we often omit sets and subscripts; for example, we write $\sum_{x}$ for $\sum_{x\in X}$ and P(x) for $P_{X}\left(x\right)$. The Rényi entropy [23] of order α is defined for positive α other than one as:

(19) $$\begin{array}{c} H_{\alpha }\left(X\right)≜\frac{1}{1-\alpha }\log\sum_{x}P{\left(x\right)}^{\alpha }. \end{array}$$

In the limit as α tends to one, the Shannon entropy is recovered, i.e., $\lim_{\alpha \to 1}H_{\alpha }\left(X\right)=H\left(X\right)$. The Arimoto–Rényi conditional entropy [24] of order α is defined for positive α other than one as:

(20) $$\begin{array}{c} H_{\alpha }\left(X|Y\right)≜\frac{\alpha }{1-\alpha }\log\sum_{y}{\left[\sum_{x}P{\left(x,y\right)}^{\alpha }\right]}^{\frac{1}{\alpha }}. \end{array}$$

In the limit as α tends to one, the Shannon conditional entropy is recovered, i.e., $\lim_{\alpha \to 1}H_{\alpha }\left(X|Y\right)=H\left(X|Y\right)$. The properties of the Arimoto–Rényi conditional entropy were studied in [7,24,25].

In the rest of this section, we recall some properties of the Arimoto–Rényi conditional entropy that will be used in Section 4 (Lemmas 1–3), and we prove auxiliary results for Section 5 (Lemmas 4–7).

**Lemma** **1**  ([7], Theorem 2)**.** *Let α>0, and let $P_{XYZ}$ be a PMF over the finite set X×Y×Z. Then,*

(21) $$\begin{array}{c} H_{\alpha }\left(X|Y,Z\right)\leq H_{\alpha }\left(X|Z\right) \end{array}$$

*with equality if and only if X⊸−Z⊸−Y form a Markov chain.*

**Lemma** **2**  ([7], Proposition 4)**.** *Let α>0, and let $P_{XYZ}$ be a PMF over the finite set X×Y×Z. Then,*

(22) $$\begin{array}{c} H_{\alpha }\left(X,Y|Z\right)\geq H_{\alpha }\left(X|Z\right) \end{array}$$

*with equality if and only if Y is uniquely determined by X and Z.*

**Lemma** **3**  ([7], Theorem 3)**.** *Let α>0, and let $P_{XYZ}$ be a PMF over the finite set X×Y×Z. Then,*

(23) $$\begin{array}{c} H_{\alpha }\left(X|Y,Z\right)\geq H_{\alpha }\left(X|Z\right)-\log\;\left|Y\right|. \end{array}$$

**Lemma** **4**  ([20], Problem 4.15(f))**.** *Let Y be a finite set, and let $f:Y\to R_{\geq 0}$. Then, for all p∈(0,1],*

(24) $$\begin{array}{c} {\left[\sum_{y}f\left(y\right)\right]}^{p}\leq \sum_{y}f{\left(y\right)}^{p}. \end{array}$$

**Proof.**  If $\sum_{y}f\left(y\right)=0$, then (24) holds because the left-hand side (LHS) and the right-hand side (RHS) are both zero. If $\sum_{y}f\left(y\right)>0$, then:

(25) $$\begin{array}{cc} \sum_{y}f{\left(y\right)}^{p} & ={\left[\sum_{y^{\prime }}f\left(y^{\prime }\right)\right]}^{p}\sum_{y}{\left[\frac{f(y)}{\sum_{y^{\prime }}f\left(y^{\prime }\right)}\right]}^{p} \end{array}$$

(26) $$\begin{array}{c} \geq {\left[\sum_{y^{\prime }}f\left(y^{\prime }\right)\right]}^{p}\sum_{y}\frac{f(y)}{\sum_{y^{\prime }}f\left(y^{\prime }\right)} \end{array}$$

(27) $$\begin{array}{c} ={\left[\sum_{y^{\prime }}f\left(y^{\prime }\right)\right]}^{p}, \end{array}$$

where (26) holds because p∈(0,1] and $f\left(y\right)/\sum_{y^{\prime }}f\left(y^{\prime }\right)\in \left[0,1\right]$ for every y∈Y.  ☐

**Lemma** **5.** *Let a, b, and c be nonnegative integers. Then, for all p>0,*

(28) $$\begin{array}{c} {\left(1+a+b+c\right)}^{p}\leq 1+4^{p}\left(a^{p}+b^{p}+c^{p}\right) \end{array}$$

*(the restriction to integers cannot be omitted; for example,* (28) *does not hold if a=b=c=0.1 and p=2).*

**Proof.**  If p∈(0,1], then (28) follows from Lemma 4 because $4^{p}\geq 1$. If p>1, then the cases with a+b+c∈{0,1,2} can be checked individually. For a+b+c≥3,

(29) $$\begin{array}{cc} {\left(1+a+b+c\right)}^{p} & ={\left[\frac{3}{a+b+c}+3\right]}^{p}\cdot {\left[\frac{a+b+c}{3}\right]}^{p} \end{array}$$

(30) $$\begin{array}{c} \leq 4^{p}\cdot {\left[\frac{a+b+c}{3}\right]}^{p} \end{array}$$

(31) $$\begin{array}{cc} & \leq 4^{p}\cdot \frac{a^{p}+b^{p}+c^{p}}{3} \end{array}$$

(32) $$\begin{array}{c} \leq 1+4^{p}\left(a^{p}+b^{p}+c^{p}\right), \end{array}$$

where (30) holds because a+b+c≥3, and (31) follows from Jensen’s inequality because $z\mapsto z^{p}$ is convex on $R_{\geq 0}$ since p>1.  ☐

**Lemma** **6.** *Let a, b, c, and d be nonnegative real numbers. Then, for all p>0,*

(33) $$\begin{array}{c} {\left(a+b+c+d\right)}^{p}\leq 4^{p}\left(a^{p}+b^{p}+c^{p}+d^{p}\right). \end{array}$$

**Proof.**  If p∈(0,1], then (33) follows from Lemma 4 because $4^{p}\geq 1$. If p>1, then:

(34) $$\begin{array}{cc} {\left(a+b+c+d\right)}^{p} & =4^{p}\cdot {\left[\frac{a+b+c+d}{4}\right]}^{p} \end{array}$$

(35) $$\begin{array}{c} \leq 4^{p}\cdot \frac{a^{p}+b^{p}+c^{p}+d^{p}}{4} \end{array}$$

(36) $$\begin{array}{c} \leq 4^{p}\left(a^{p}+b^{p}+c^{p}+d^{p}\right), \end{array}$$

where (35) follows from Jensen’s inequality because $z\mapsto z^{p}$ is convex on $R_{\geq 0}$ since p>1. ☐

**Lemma** **7**  (Rosenthal)**.** *Let p>1, and let $X_{1},\ldots ,X_{n}$ be independent random variables that are either zero or one. Then, $X≜\sum_{i=1}^{n}X_{i}$ satisfies:*

(37) $$\begin{array}{c} E\left[X^{p}\right]\leq 2^{p^{2}}\max\left\{E\left[X\right],\;E{\left[X\right]}^{p}\right\}. \end{array}$$

**Proof.**  This is a special case of [11] (Lemma 1). For convenience, we also provide a self-contained proof:

(38) $$\begin{array}{cc} E[X^{p}] & =E\left[\;\sum_{i\in \{1,\ldots ,n\}}X_{i}\cdot {\left\{\;\sum_{j\in \{1,\ldots ,n\}}X_{j}\right\}}^{p-1}\;\right] \end{array}$$

(39) $$\begin{array}{cc} & =E\left[\;\sum_{i\in \{1,\ldots ,n\}}X_{i}\cdot {\left\{1+\sum_{j\in \{1,\ldots ,n\}\setminus \{i\}}X_{j}\right\}}^{p-1}\;\right] \end{array}$$

(40) $$\begin{array}{cc} & =\sum_{i\in \{1,\ldots ,n\}}E\left[X_{i}\cdot {\left\{1+\sum_{j\in \{1,\ldots ,n\}\setminus \{i\}}X_{j}\right\}}^{p-1}\;\right] \end{array}$$

(41) $$\begin{array}{cc} & =\sum_{i\in \{1,\ldots ,n\}}E\left[X_{i}\right]\cdot E\left[{\left\{1+\sum_{j\in \{1,\ldots ,n\}\setminus \{i\}}X_{j}\right\}}^{p-1}\;\right] \end{array}$$

(42) $$\begin{array}{cc} & \leq \sum_{i\in \{1,\ldots ,n\}}E\left[X_{i}\right]\cdot E\left[{\left\{1+\sum_{j\in \{1,\ldots ,n\}}X_{j}\right\}}^{p-1}\;\right] \end{array}$$

(43) $$\begin{array}{cc} & =E\left[X\right]\cdot E[{\left(1+X\right)}^{p-1}] \end{array}$$

(44) $$\begin{array}{cc} & \leq E\left[X\right]\cdot 2^{p-1}\cdot \left(1+E\left[X^{p-1}\right]\right) \end{array}$$

(45) $$\begin{array}{cc} & =2^{p-1}\left(E\left[X\right]+E\left[X\right]\;E\left[X^{p-1}\right]\right) \end{array}$$

(46) $$\begin{array}{cc} & \leq 2^{p-1}\left(E\left[X\right]+E\left[X\right]\;E{\left[X^{p}\right]}^{\frac{p-1}{p}}\right) \end{array}$$

(47) $$\begin{array}{cc} & \leq 2^{p}\max\left\{E\left[X\right],\;E\left[X\right]\;E{\left[X^{p}\right]}^{\frac{p-1}{p}}\right\}, \end{array}$$

where (39) holds because each $X_{i}$ is either zero or one; (41) holds because $X_{1},\ldots ,X_{n}$ are independent; (42) holds because $z\mapsto z^{p-1}$ is increasing on $R_{\geq 0}$ for p>1; (44) holds because for real numbers a≥0, b≥0, and r>0, we have ${\left(a+b\right)}^{r}\leq {\left(2\;\max\left\{a,b\right\}\right)}^{r}=2^{r}\max\left\{a^{r},b^{r}\right\}\leq 2^{r}\left(a^{r}+b^{r}\right)$; and (46) follows from Jensen’s inequality because $z\mapsto z^{(p-1)/p}$ is concave on $R_{\geq 0}$ for p>1.

We now consider two cases depending on which term on the RHS of (47) achieves the maximum: If the maximum is achieved by E[X], then $E\left[X^{p}\right]\leq 2^{p}\;E\left[X\right]$, which implies (37) because $2^{p}\leq 2^{p^{2}}$ since p>1. If the maximum is achieved by $E\left[X\right]\;E{\left[X^{p}\right]}^{(p-1)/p}$, then:

(48) $$\begin{array}{c} E\left[X^{p}\right]\leq 2^{p}\;E\left[X\right]\;E{\left[X^{p}\right]}^{\frac{p-1}{p}}. \end{array}$$

Rearranging (48), we obtain:

(49) $$\begin{array}{c} E\left[X^{p}\right]\leq 2^{p^{2}}\;E{\left[X\right]}^{p}, \end{array}$$

so (37) holds also in this case. ☐

## 4. Converse

In this section, we prove a nonasymptotic and an asymptotic converse result (Theorem 2 and Corollary 1, respectively).

**Theorem** **2.** *Let U⊸−X⊸−Y⊸−V form a Markov chain over the finite set U×X×Y×V, and let τ≜1+ln|X×Y|. Then, for every ρ>0 and for every guesser, the ρth moment of the number of guesses it takes to guess the pair (X,Y) based on the side information (U,V) satisfies:*

(50) $$\begin{array}{cc} E[G{\left(X,Y|U,V\right)}^{\rho }]\geq \max\{ & 2^{\rho (H_{\tilde{\rho }}\left(X|Y\right)-\log\;\left|U\right|-\log\tau )},\\ & 2^{\rho (H_{\tilde{\rho }}\left(Y|X\right)-\log\;\left|V\right|-\log\tau )},\\ & 2^{\rho (H_{\tilde{\rho }}\left(X,Y\right)-\log\;\left|U\times V\right|-\log\tau )}\}. \end{array}$$

**Proof.**  We view (50) as three lower bounds corresponding to the three terms in the maximization on its RHS. The lower bound involving $H_{\tilde{\rho }}\left(X,Y\right)$ holds because:

(51) $$\begin{array}{cc} E[G{\left(X,Y|U,V\right)}^{\rho }] & \geq 2^{\rho (H_{\tilde{\rho }}\left(X,Y|U,V\right)-\log\tau )} \end{array}$$

(52) $$\begin{array}{cc} \; & \geq 2^{\rho (H_{\tilde{\rho }}\left(X,Y\right)-\log\;\left|U\times V\right|-\log\tau )}, \end{array}$$

where (51) follows from (18) and (52) follows from Lemma 3. The lower bound involving $H_{\tilde{\rho }}\left(X|Y\right)$ holds because:

(53) $$\begin{array}{cc} E[G{\left(X,Y|U,V\right)}^{\rho }] & \geq 2^{\rho (H_{\tilde{\rho }}\left(X,Y|U,V\right)-\log\tau )} \end{array}$$

(54) $$\begin{array}{cc} \; & \geq 2^{\rho (H_{\tilde{\rho }}\left(X,Y|U,V,Y\right)-\log\tau )} \end{array}$$

(55) $$\begin{array}{cc} \; & =2^{\rho (H_{\tilde{\rho }}\left(X|U,V,Y\right)-\log\tau )} \end{array}$$

(56) $$\begin{array}{cc} \; & =2^{\rho (H_{\tilde{\rho }}\left(X|U,Y\right)-\log\tau )} \end{array}$$

(57) $$\begin{array}{cc} \; & \geq 2^{\rho (H_{\tilde{\rho }}\left(X|Y\right)-\log\;\left|U\right|-\log\tau )}, \end{array}$$

where (53) follows from (18); (54) follows from Lemma 1; (55) follows from Lemma 2; (56) follows from Lemma 1 because X⊸−(U,Y)⊸−V form a Markov chain; and (57) follows from Lemma 3. The lower bound involving $H_{\tilde{\rho }}\left(Y|X\right)$ is analogous to the one with $H_{\tilde{\rho }}\left(X|Y\right)$. ☐

**Corollary** **1.** *For any ρ>0, rate pairs outside R(ρ) are not achievable.*

**Proof.**  We first show that (8) is necessary for a rate pair $\left(R_{X},R_{Y}\right)\in R_{\geq 0}^{2}$ to be achievable. Indeed, if (8) does not hold, then there exists an ϵ>0 such that for infinitely many *n*,

(58) $$\begin{array}{c} \frac{H_{\tilde{\rho }}\left(X^{n},Y^{n}\right)}{n}\geq R_{X}+R_{Y}+\epsilon . \end{array}$$

Using Theorem 2 with $X^{\prime }≜X^{n}$, $Y^{\prime }≜Y^{n}$, $U≜\{1,\ldots ,\left\lfloor 2^{nR_{X}}\right\rfloor \}$, $V≜\{1,\ldots ,\left\lfloor 2^{nR_{Y}}\right\rfloor \}$, $P_{X^{\prime }Y^{\prime }}≜P_{X^{n}Y^{n}}$, $U≜f_{n}\left(X^{n}\right)$, $V≜g_{n}\left(Y^{n}\right)$, and $\tau_{n}=1+n\ln\;\left|X\times Y\right|$ leads to:

(59) $$\begin{array}{cc} E[G{\left(X^{n},Y^{n}|U,V\right)}^{\rho }] & \geq 2^{\rho (H_{\tilde{\rho }}\left(X^{n},Y^{n}\right)-\log\;\left|U\times V\right|-\log\tau_{n})} \end{array}$$

(60) $$\begin{array}{cc} \; & \geq 2^{\rho n(\frac{1}{n}H_{\tilde{\rho }}\left(X^{n},Y^{n}\right)-R_{X}-R_{Y}-\frac{1}{n}\log\tau_{n})}. \end{array}$$

It follows from (60), (58), and the fact that $\frac{1}{n}\log\tau_{n}$ tends to zero as *n* tends to infinity that the LHS of (59) cannot tend to one as *n* tends to infinity, so $\left(R_{X},R_{Y}\right)$ is not achievable if (8) does not hold. The necessity of (6) and (7) can be shown in the same way. ☐

## 5. Achievability

In this section, we prove a nonasymptotic and an asymptotic achievability result (Theorem 3 and Corollary 2, respectively).

**Theorem** **3.** *Let X, Y, U, and V be finite nonempty sets; let $P_{XY}$ be a PMF; let ρ>0; and let ϵ>0 be such that:*

(61) $$\begin{array}{cc} \log\;|U| & \geq H_{\tilde{\rho }}\left(X|Y\right)+\epsilon , \end{array}$$

(62) $$\begin{array}{cc} \log\;|V| & \geq H_{\tilde{\rho }}\left(Y|X\right)+\epsilon , \end{array}$$

(63) $$\begin{array}{cc} \log\;|U\times V| & \geq H_{\tilde{\rho }}\left(X,Y\right)+\epsilon . \end{array}$$

*Then, there exist functions f:X→U and g:Y→V and a guesser such that the ρth moment of the number of guesses needed to guess the pair (X,Y) based on the side information (f(X),g(Y)) satisfies:*

(64) $$\begin{array}{cc} E\left[G{\left(X,Y|f(X),g(Y)\right)}^{\rho }\right] & \leq \left\{\begin{array}{cc} 1+4^{\rho +1}\cdot 2^{-\rho \epsilon } & \mathrm{if}\;\rho \in (0,1],\\ 1+4^{{\left(\rho +1\right)}^{2}}\cdot 2^{-\epsilon } & \mathrm{if}\;\rho >1. \end{array}\right. \end{array}$$

**Proof.**  Our achievability result relies on random binning: we map each x∈X uniformly at random to some u∈U and each y∈Y uniformly at random to some v∈V. We then show that the ρth moment of the number of guesses averaged over all such mappings f:X→U and g:Y→V is upper bounded by the RHS of (64). From this, we conclude that there exist *f* and *g* that satisfy (64).

Let the guessing function *G* correspond to guessing in decreasing order of probability [2] (ties can be resolved arbitrarily). Let *f* and *g* be distributed as described above, and denote by $E_{f\;,\;g}\left[\cdot \right]$ the expectation with respect to *f* and *g*. Then,

(65) $$\begin{array}{cc} E_{f\;,\;g}\left[E[G{\left(X,Y|f\left(X\right),g\left(Y\right)\right)}^{\rho }]\right] & =\sum_{x,y}P\left(x,y\right)\;E_{f\;,\;g}\left[G{\left(x,y|f\left(x\right),g\left(y\right)\right)}^{\rho }\right] \end{array}$$

(66) $$\begin{array}{cc} \; & \leq \sum_{x,y}P\left(x,y\right)\;E_{f\;,\;g}\left[{\left\{\sum_{x^{\prime },y^{\prime }}\psi \left(x^{\prime },y^{\prime }\right)\phi_{f}\left(x^{\prime }\right)\phi_{g}\left(y^{\prime }\right)\right\}}^{\rho }\;\right] \end{array}$$

(67) $$\begin{array}{cc} \; & =\sum_{x,y}P\left(x,y\right)\;E_{f\;,\;g}\left[{\left(1+\beta_{1}+\beta_{2}+\beta_{3}\right)}^{\rho }\right] \end{array}$$

(68) $$\begin{array}{cc} \; & \leq 1+4^{\rho }\sum_{x,y}P\left(x,y\right)\left(E_{f\;,\;g}\left[\beta_{1}^{\rho }\right]+E_{f\;,\;g}\left[\beta_{2}^{\rho }\right]+E_{f\;,\;g}\left[\beta_{3}^{\rho }\right]\right) \end{array}$$

with:

(69) $$\begin{array}{cc} \psi \left(x^{\prime },y^{\prime }\right)=\psi \left(x,y,x^{\prime },y^{\prime }\right) & ≜𝟙\{P\left(x^{\prime },y^{\prime }\right)\geq P\left(x,y\right)\}, \end{array}$$

(70) $$\begin{array}{cc} \phi_{f}\left(x^{\prime }\right)=\phi_{f}\left(x,x^{\prime }\right) & ≜𝟙\{f\left(x^{\prime }\right)=f\left(x\right)\}, \end{array}$$

(71) $$\begin{array}{cc} \phi_{g}\left(y^{\prime }\right)=\phi_{g}\left(y,y^{\prime }\right) & ≜𝟙\{g\left(y^{\prime }\right)=g\left(y\right)\}, \end{array}$$

(72) $$\begin{array}{cc} \beta_{1}=\beta_{1}\left(x,y,f\right) & ≜\sum_{x^{\prime }\neq x}\psi \left(x^{\prime },y\right)\phi_{f}\left(x^{\prime }\right), \end{array}$$

(73) $$\begin{array}{cc} \beta_{2}=\beta_{2}\left(x,y,g\right) & ≜\sum_{y^{\prime }\neq y}\psi \left(x,y^{\prime }\right)\phi_{g}\left(y^{\prime }\right), \end{array}$$

(74) $$\begin{array}{cc} \beta_{3}=\beta_{3}\left(x,y,f,g\right) & ≜\sum_{x^{\prime }\neq x,y^{\prime }\neq y}\psi \left(x^{\prime },y^{\prime }\right)\phi_{f}\left(x^{\prime }\right)\phi_{g}\left(y^{\prime }\right), \end{array}$$

where 𝟙{·} is the indicator function that is one if the condition comprising its argument is true and zero otherwise; (65) holds because (f,g) and (X,Y) are independent; (66) holds because the number of guesses is upper bounded by the number of $\left(x^{\prime },y^{\prime }\right)$ that are at least as likely as (x,y) and that are mapped to the same labels (u,v) as (x,y); (67) follows from splitting the sum depending on whether $x^{\prime }=x$ or not and whether $y^{\prime }=y$ or not and from the fact that $\psi \left(x,y\right)=\phi_{f}\left(x\right)=\phi_{g}\left(y\right)=1$; and (68) follows from Lemma 5 because $\beta_{1}$, $\beta_{2}$, and $\beta_{3}$ are nonnegative integers. As indicated in (69)–(74), the dependence of ψ, $\phi_{f}$, $\phi_{g}$, $\beta_{1}$, $\beta_{2}$, and $\beta_{3}$ on *x*, *y*, *f*, and *g* is implicit in our notation.

We first treat the case ρ∈(0,1]. We bound the terms on the RHS of (68) as follows:

(75) $$\begin{array}{cc} \sum_{x,y}P\left(x,y\right)\;E_{f\;,\;g}\left[\beta_{1}^{\rho }\right] & \leq \sum_{x,y}P\left(x,y\right)\;E_{f\;,\;g}{\left[\beta_{1}\right]}^{\rho } \end{array}$$

(76) $$\begin{array}{cc} \; & =\sum_{x,y}P\left(x,y\right){\left[\;\sum_{x^{\prime }\neq x}\psi \left(x^{\prime },y\right)\;\frac{1}{\left|U\right|}\right]}^{\rho } \end{array}$$

(77) $$\begin{array}{cc} \; & \leq \sum_{x,y}P\left(x,y\right){\left[\sum_{x^{\prime }}{\left[\frac{P(x^{\prime },y)}{P(x,y)}\right]}^{\tilde{\rho }}\;\frac{1}{\left|U\right|}\right]}^{\rho } \end{array}$$

(78) $$\begin{array}{c} \;=\frac{1}{{\left|U\right|}^{\rho }}\;\sum_{x,y}P{\left(x,y\right)}^{\tilde{\rho }}{\left[\sum_{x^{\prime }}P{\left(x^{\prime },y\right)}^{\tilde{\rho }}\right]}^{\rho } \end{array}$$

(79) $$\begin{array}{c} \;=\frac{1}{{\left|U\right|}^{\rho }}\;\sum_{y}\left[\sum_{x}P{\left(x,y\right)}^{\tilde{\rho }}\right]{\left[\sum_{x^{\prime }}P{\left(x^{\prime },y\right)}^{\tilde{\rho }}\right]}^{\rho } \end{array}$$

(80) $$\begin{array}{c} \;=\frac{1}{{\left|U\right|}^{\rho }}\;\sum_{y}{\left[\sum_{x}P{\left(x,y\right)}^{\tilde{\rho }}\right]}^{1+\rho } \end{array}$$

(81) $$\begin{array}{cc} \; & =2^{\rho (H_{\tilde{\rho }}\left(X|Y\right)-\log\;\left|U\right|)} \end{array}$$

(82) $$\begin{array}{cc} \; & \leq 2^{-\rho \epsilon }, \end{array}$$

where (75) follows from Jensen’s inequality because $z\mapsto z^{\rho }$ is concave on $R_{\geq 0}$ since ρ∈(0,1]; (76) holds because the expectation operator is linear and because $E_{f\;,\;g}\left[\phi_{f}\left(x^{\prime }\right)\right]=1/\left|U\right|$ since $x^{\prime }\neq x$; in (77), we extended the inner summation and used that $\psi \left(x^{\prime },y\right)\leq {\left[P\left(x^{\prime },y\right)/P\left(x,y\right)\right]}^{\tilde{\rho }}$; and (82) follows from (61). In the same way, we obtain:

(83) $$\begin{array}{c} \sum_{x,y}P\left(x,y\right)\;E_{f\;,\;g}\left[\beta_{2}^{\rho }\right]\leq 2^{-\rho \epsilon }. \end{array}$$

Similarly,

(84) $$\begin{array}{cc} \sum_{x,y}P\left(x,y\right)\;E_{f\;,\;g}\left[\beta_{3}^{\rho }\right] & \leq \sum_{x,y}P\left(x,y\right)\;E_{f\;,\;g}{\left[\beta_{3}\right]}^{\rho } \end{array}$$

(85) $$\begin{array}{cc} \; & =\sum_{x,y}P\left(x,y\right){\left[\;\sum_{x^{\prime }\neq x,y^{\prime }\neq y}\psi \left(x^{\prime },y^{\prime }\right)\frac{1}{\left|U\times V\right|}\right]}^{\rho } \end{array}$$

(86) $$\begin{array}{c} \;\leq \sum_{x,y}P\left(x,y\right){\left[\;\sum_{x^{\prime },y^{\prime }}{\left[\frac{P(x^{\prime },y^{\prime })}{P(x,y)}\right]}^{\tilde{\rho }}\frac{1}{\left|U\times V\right|}\right]}^{\rho } \end{array}$$

(87) $$\begin{array}{c} \;=\frac{1}{{\left|U\times V\right|}^{\rho }}\sum_{x,y}P{\left(x,y\right)}^{\tilde{\rho }}{\left[\;\sum_{x^{\prime },y^{\prime }}P{\left(x^{\prime },y^{\prime }\right)}^{\tilde{\rho }}\right]}^{\rho } \end{array}$$

(88) $$\begin{array}{c} \;=\frac{1}{{\left|U\times V\right|}^{\rho }}{\left[\sum_{x,y}P{\left(x,y\right)}^{\tilde{\rho }}\right]}^{1+\rho } \end{array}$$

(89) $$\begin{array}{cc} \; & =2^{\rho (H_{\tilde{\rho }}\left(X,Y\right)-\log\;\left|U\times V\right|)} \end{array}$$

(90) $$\begin{array}{cc} \; & \leq 2^{-\rho \epsilon }. \end{array}$$

From (68), (82), (83), and (90), we obtain:

(91) $$\begin{array}{cc} E_{f\;,\;g}\left[E[G{\left(X,Y|f\left(X\right),g\left(Y\right)\right)}^{\rho }]\right] & \leq 1+3\cdot 4^{\rho }\cdot 2^{-\rho \epsilon } \end{array}$$

(92) $$\begin{array}{c} \;\leq 1+4^{\rho +1}\cdot 2^{-\rho \epsilon } \end{array}$$

and hence infer the existence of f: X→U and g: Y→V satisfying (64).

We now consider (68) when ρ>1. Unlike in the case ρ∈(0,1], we cannot use Jensen’s inequality as we did in (75). Instead, for fixed x∈X and y∈Y, we upper-bound the first expectation on the RHS of (68) by:

(93) $$\begin{array}{cc} E_{f\;,\;g}\left[\beta_{1}^{\rho }\right] & \leq 2^{\rho^{2}}\max\left\{E_{f\;,\;g}\left[\beta_{1}\right],\;E_{f\;,\;g}{\left[\beta_{1}\right]}^{\rho }\right\} \end{array}$$

(94) $$\begin{array}{cc} \; & \leq 2^{\rho^{2}}\left(E_{f\;,\;g}{\left[\beta_{1}\right]}^{\rho }+E_{f\;,\;g}\left[\beta_{1}\right]\right), \end{array}$$

where (93) follows from Lemma 7 because ρ>1 and because $\beta_{1}$ is a sum of independent random variables taking values in {0,1}. By the same steps as in (76)–(82),

(95) $$\begin{array}{c} \sum_{x,y}P\left(x,y\right)\;E_{f\;,\;g}{\left[\beta_{1}\right]}^{\rho }\leq 2^{-\rho \epsilon }. \end{array}$$

As to the expectation of the other term on the RHS of (94),

(96) $$\begin{array}{cc} \sum_{x,y}P\left(x,y\right)\;E_{f\;,\;g}\left[\beta_{1}\right] & \leq {\left[\sum_{x,y}P\left(x,y\right)\;E_{f\;,\;g}{\left[\beta_{1}\right]}^{\rho }\right]}^{\frac{1}{\rho }} \end{array}$$

(97) $$\begin{array}{cc} & \leq 2^{-\epsilon }, \end{array}$$

where (96) follows from Jensen’s inequality because $z\mapsto z^{\frac{1}{\rho }}$ is concave on $R_{\geq 0}$ since ρ>1, and (97) follows from (95). From (94), (95), and (97), we obtain:

(98) $$\begin{array}{cc} \sum_{x,y}P\left(x,y\right)\;E_{f\;,\;g}\left[\beta_{1}^{\rho }\right] & \leq 2^{\rho^{2}}\left(2^{-\rho \epsilon }+2^{-\epsilon }\right) \end{array}$$

(99) $$\begin{array}{c} \;\leq 2^{\rho^{2}+1}\cdot 2^{-\epsilon }, \end{array}$$

where (99) holds because $2^{-\rho \epsilon }\leq 2^{-\epsilon }$ since ρ>1 and ϵ>0. In the same way, we obtain for the second expectation on the RHS of (68):

(100) $$\begin{array}{c} \sum_{x,y}P\left(x,y\right)\;E_{f\;,\;g}\left[\beta_{2}^{\rho }\right]\leq 2^{\rho^{2}+1}\cdot 2^{-\epsilon }. \end{array}$$

Bounding $E_{f\;,\;g}\left[\beta_{3}^{\rho }\right]$, i.e., the third expectation on the RHS of (68), is more involved because $\beta_{3}$ is not a sum of independent random variables. Our approach builds on the ideas used by Rosenthal [11] (Proof of Lemma 1); compare (47) and (48) with (108) and (123) ahead. For fixed x∈X and y∈Y,

(101) $$\begin{array}{cc} E_{f\;,\;g}\left[\beta_{3}^{\rho }\right] & =E_{f\;,\;g}\left[\;\sum_{x^{\prime }\neq x,y^{\prime }\neq y}\psi \left(x^{\prime },y^{\prime }\right)\phi_{f}\left(x^{\prime }\right)\phi_{g}\left(y^{\prime }\right)\cdot {\left\{\sum_{\tilde{x}\neq x,\tilde{y}\neq y}\psi \left(\tilde{x},\tilde{y}\right)\phi_{f}\left(\tilde{x}\right)\phi_{g}\left(\tilde{y}\right)\right\}}^{\rho -1}\;\right] \end{array}$$

(102) $$\begin{array}{c} \;=E_{f\;,\;g}\left[\;\sum_{x^{\prime }\neq x,y^{\prime }\neq y}\psi \left(x^{\prime },y^{\prime }\right)\phi_{f}\left(x^{\prime }\right)\phi_{g}\left(y^{\prime }\right)\cdot {\left(1+\gamma_{1}+\gamma_{2}+\gamma_{3}\right)}^{\rho -1}\right] \end{array}$$

(103) $$\begin{array}{c} \;=\sum_{x^{\prime }\neq x,y^{\prime }\neq y}E_{f\;,\;g}\left[\psi \left(x^{\prime },y^{\prime }\right)\phi_{f}\left(x^{\prime }\right)\phi_{g}\left(y^{\prime }\right)\cdot {\left(1+\gamma_{1}+\gamma_{2}+\gamma_{3}\right)}^{\rho -1}\right] \end{array}$$

(104) $$\begin{array}{cc} \; & =\sum_{x^{\prime }\neq x,y^{\prime }\neq y}E_{f\;,\;g}\left[\psi \left(x^{\prime },y^{\prime }\right)\phi_{f}\left(x^{\prime }\right)\phi_{g}\left(y^{\prime }\right)\right]\cdot E_{f\;,\;g}\left[{\left(1+\gamma_{1}+\gamma_{2}+\gamma_{3}\right)}^{\rho -1}\right] \end{array}$$

(105) $$\begin{array}{c} \leq \sum_{x^{\prime }\neq x,y^{\prime }\neq y}E_{f\;,\;g}\left[\psi \left(x^{\prime },y^{\prime }\right)\phi_{f}\left(x^{\prime }\right)\phi_{g}\left(y^{\prime }\right)\right]\cdot E_{f\;,\;g}\left[{\left(1+\delta_{1}+\delta_{2}+\beta_{3}\right)}^{\rho -1}\right] \end{array}$$

(106) $$\begin{array}{cc} \; & \leq \sum_{x^{\prime }\neq x,y^{\prime }\neq y}E_{f\;,\;g}\left[\psi \left(x^{\prime },y^{\prime }\right)\phi_{f}\left(x^{\prime }\right)\phi_{g}\left(y^{\prime }\right)\right]\cdot 4^{\rho -1}\cdot E_{f\;,\;g}\left[1+\delta_{1}^{\rho -1}+\delta_{2}^{\rho -1}+\beta_{3}^{\rho -1}\right] \end{array}$$

$$\begin{array}{c} \;=4^{\rho -1}\{E_{f\;,\;g}\left[\beta_{3}\right]+\sum_{y^{\prime }\neq y}\frac{1}{\left|V\right|}\;E_{f\;,\;g}\left[\delta_{1}\right]\;E_{f\;,\;g}\left[\delta_{1}^{\rho -1}\right] \end{array}$$

(107) =4ρ−1{+∑x′≠x1|U|Ef,g[δ2]Ef,g[δ2ρ−1]+Ef,g[β3]Ef,g[β3ρ−1]}

$$\begin{array}{c} \;\leq 4^{\rho }\max\{E_{f\;,\;g}\left[\beta_{3}\right],\;\sum_{y^{\prime }\neq y}\frac{1}{\left|V\right|}\;E_{f\;,\;g}\left[\delta_{1}\right]\;E_{f\;,\;g}\left[\delta_{1}^{\rho -1}\right], \end{array}$$

(108) ≤4ρmax{∑x′≠x1|U|Ef,g[δ2]Ef,g[δ2ρ−1],Ef,g[β3]Ef,g[β3ρ−1]}

with:

(109) $$\begin{array}{cc} \gamma_{1}=\gamma_{1}\left(x,y,x^{\prime },y^{\prime },f\right) & ≜\sum_{\tilde{x}\notin \left\{x,x^{\prime }\right\}}\psi \left(\tilde{x},y^{\prime }\right)\phi_{f}\left(\tilde{x}\right), \end{array}$$

(110) $$\begin{array}{cc} \gamma_{2}=\gamma_{2}\left(x,y,x^{\prime },y^{\prime },g\right) & ≜\sum_{\tilde{y}\notin \left\{y,y^{\prime }\right\}}\psi \left(x^{\prime },\tilde{y}\right)\phi_{g}\left(\tilde{y}\right), \end{array}$$

(111) $$\begin{array}{cc} \gamma_{3}=\gamma_{3}\left(x,y,x^{\prime },y^{\prime },f,g\right) & ≜\sum_{\tilde{x}\notin \left\{x,x^{\prime }\right\},\tilde{y}\notin \left\{y,y^{\prime }\right\}}\psi \left(\tilde{x},\tilde{y}\right)\phi_{f}\left(\tilde{x}\right)\phi_{g}\left(\tilde{y}\right), \end{array}$$

(112) $$\begin{array}{cc} \delta_{1}=\delta_{1}\left(x,y,y^{\prime },f\right) & ≜\sum_{\tilde{x}\neq x}\psi \left(\tilde{x},y^{\prime }\right)\phi_{f}\left(\tilde{x}\right), \end{array}$$

(113) $$\begin{array}{cc} \delta_{2}=\delta_{2}\left(x,y,x^{\prime },g\right) & ≜\sum_{\tilde{y}\neq y}\psi \left(x^{\prime },\tilde{y}\right)\phi_{g}\left(\tilde{y}\right), \end{array}$$

where (102) follows from splitting the sum in braces depending on whether $\tilde{x}=x^{\prime }$ or not and whether $\tilde{y}=y^{\prime }$ or not and from assuming $\psi \left(x^{\prime },y^{\prime }\right)=\phi_{f}\left(x^{\prime }\right)=\phi_{g}\left(y^{\prime }\right)=1$ within the braces, which does not change the value of the expression because it is multiplied by $\psi \left(x^{\prime },y^{\prime }\right)\phi_{f}\left(x^{\prime }\right)\phi_{g}\left(y^{\prime }\right)$; (104) holds because $\left(\phi_{f}\left(x^{\prime }\right),\phi_{g}\left(y^{\prime }\right)\right)$ and $\left(\gamma_{1},\gamma_{2},\gamma_{3}\right)$ are independent since $\tilde{x}\neq x^{\prime }$ and $\tilde{y}\neq y^{\prime }$; (105) holds because ρ−1>0, $\gamma_{1}\leq \delta_{1}$, $\gamma_{2}\leq \delta_{2}$, and $\gamma_{3}\leq \beta_{3}$; (106) follows from Lemma 6; and (107) follows from identifying $E_{f\;,\;g}\left[\beta_{3}\right]$, $E_{f\;,\;g}\left[\delta_{1}\right]$, and $E_{f\;,\;g}\left[\delta_{2}\right]$ because $\phi_{f}\left(x^{\prime }\right)$ and $\phi_{g}\left(y^{\prime }\right)$ are independent, $E_{f\;,\;g}\left[\phi_{f}\left(x^{\prime }\right)\right]=1/\left|U\right|$, and $E_{f\;,\;g}\left[\phi_{g}\left(y^{\prime }\right)\right]=1/\left|V\right|$. As indicated in (109)–(113), the dependence of $\gamma_{1}$, $\gamma_{2}$, $\gamma_{3}$, $\delta_{1}$, and $\delta_{2}$ on *x*, *y*, $x^{\prime }$, $y^{\prime }$, *f*, and *g* is implicit in our notation.

To bound $E_{f\;,\;g}\left[\beta_{3}^{\rho }\right]$ further, we study some of the terms on the RHS of (108) separately, starting with the second, which involves the sum over $y^{\prime }$. For fixed x∈X, y∈Y, and $y^{\prime }\in Y\setminus \left\{y\right\}$,

(114) $$\begin{array}{cc} E_{f\;,\;g}\left[\delta_{1}\right]\;E_{f\;,\;g}\left[\delta_{1}^{\rho -1}\right] & \leq E_{f\;,\;g}{\left[\delta_{1}^{\rho }\right]}^{\frac{1}{\rho }}\;E_{f\;,\;g}{\left[\delta_{1}^{\rho }\right]}^{\frac{\rho -1}{\rho }} \end{array}$$

(115) $$\begin{array}{c} =E_{f\;,\;g}\left[\delta_{1}^{\rho }\right] \end{array}$$

(116) $$\begin{array}{cc} & \leq 2^{\rho^{2}}\max\left\{E_{f\;,\;g}\left[\delta_{1}\right],\;E_{f\;,\;g}{\left[\delta_{1}\right]}^{\rho }\right\} \end{array}$$

(117) $$\begin{array}{c} \leq 2^{\rho^{2}}\left(E_{f\;,\;g}\left[\delta_{1}\right]+E_{f\;,\;g}{\left[\delta_{1}\right]}^{\rho }\right), \end{array}$$

where (114) follows from Jensen’s inequality because $z\mapsto z^{\frac{1}{\rho }}$ and $z\mapsto z^{\frac{\rho -1}{\rho }}$ are both concave on $R_{\geq 0}$ since ρ>1, and (116) follows from Lemma 7 because ρ>1 and because $\delta_{1}$ is a sum of independent random variables taking values in {0,1}. This implies that for fixed x∈X and y∈Y,

(118) $$\begin{array}{cc} \sum_{y^{\prime }\neq y}\frac{1}{\left|V\right|}\;E_{f\;,\;g}\left[\delta_{1}\right]\;E_{f\;,\;g}\left[\delta_{1}^{\rho -1}\right] & \leq 2^{\rho^{2}}\sum_{y^{\prime }\neq y}\frac{1}{\left|V\right|}\left(E_{f\;,\;g}\left[\delta_{1}\right]+E_{f\;,\;g}{\left[\delta_{1}\right]}^{\rho }\right) \end{array}$$

(119) $$\begin{array}{cc} \; & =2^{\rho^{2}}\;E_{f\;,\;g}\left[\beta_{3}\right]+2^{\rho^{2}}\sum_{y^{\prime }\neq y}\frac{1}{\left|V\right|}\;E_{f\;,\;g}{\left[\delta_{1}\right]}^{\rho }, \end{array}$$

where (119) follows from the definitions of $\delta_{1}$ and $\beta_{3}$. Similarly, for the third term on the RHS of (108),

(120) $$\begin{array}{cc} \sum_{x^{\prime }\neq x}\frac{1}{\left|U\right|}\;E_{f\;,\;g}\left[\delta_{2}\right]\;E_{f\;,\;g}\left[\delta_{2}^{\rho -1}\right] & \leq 2^{\rho^{2}}\;E_{f\;,\;g}\left[\beta_{3}\right]+2^{\rho^{2}}\sum_{x^{\prime }\neq x}\frac{1}{\left|U\right|}\;E_{f\;,\;g}{\left[\delta_{2}\right]}^{\rho }. \end{array}$$

With the help of (119) and (120), we now go back to (108) and argue that it implies that for fixed x∈X and y∈Y,

(121) $$E_{f\;,\;g}\left[\beta_{3}^{\rho }\right]\leq 2\cdot 4^{\rho^{2}}\;[E_{f\;,\;g}\left[\beta_{3}\right]+\sum_{y^{\prime }\neq y}\frac{1}{\left|V\right|}\;E_{f\;,\;g}{\left[\delta_{1}\right]}^{\rho }+\sum_{x^{\prime }\neq x}\frac{1}{\left|U\right|}\;E_{f\;,\;g}{\left[\delta_{2}\right]}^{\rho }+E_{f\;,\;g}{\left[\beta_{3}\right]}^{\rho }].$$

To prove this, we consider four cases depending on which term on the RHS of (108) achieves the maximum: If $E_{f\;,\;g}\left[\beta_{3}\right]$ achieves the maximum, then (121) holds because $4^{\rho }\leq 2\cdot 4^{\rho^{2}}$. If the LHS of (118) achieves the maximum, then (121) follows from (119) because $4^{\rho }\cdot 2^{\rho^{2}}\leq 2\cdot 4^{\rho^{2}}$. If the LHS of (120) achieves the maximum, then (121) follows similarly. Finally, if $E_{f\;,\;g}\left[\beta_{3}\right]\;E_{f\;,\;g}\left[\beta_{3}^{\rho -1}\right]$ achieves the maximum, then:

(122) $$\begin{array}{cc} E_{f\;,\;g}\left[\beta_{3}^{\rho }\right] & \leq 4^{\rho }\;E_{f\;,\;g}\left[\beta_{3}\right]\;E_{f\;,\;g}\left[\beta_{3}^{\rho -1}\right] \end{array}$$

(123) $$\begin{array}{c} \leq 4^{\rho }\;E_{f\;,\;g}\left[\beta_{3}\right]\;E_{f\;,\;g}{\left[\beta_{3}^{\rho }\right]}^{\frac{\rho -1}{\rho }}, \end{array}$$

where (123) follows from Jensen’s inequality because $z\mapsto z^{\frac{\rho -1}{\rho }}$ is concave on $R_{\geq 0}$ for ρ>1. Rearranging (123), we obtain:

(124) $$\begin{array}{c} E_{f\;,\;g}\left[\beta_{3}^{\rho }\right]\leq 4^{\rho^{2}}\;E_{f\;,\;g}{\left[\beta_{3}\right]}^{\rho }, \end{array}$$

so (121) holds also in this case.

Having established (121), we now take the expectation of its sides to obtain:

(125) $$\begin{array}{c} \sum_{x,y}P\left(x,y\right)\;E_{f\;,\;g}\left[\beta_{3}^{\rho }\right]\leq 2\cdot 4^{\rho^{2}}\sum_{x,y}P\left(x,y\right)\left[E_{f\;,\;g}\left[\beta_{3}\right]+\sum_{y^{\prime }\neq y}\frac{1}{\left|V\right|}\;E_{f\;,\;g}{\left[\delta_{1}\right]}^{\rho }+\sum_{x^{\prime }\neq x}\frac{1}{\left|U\right|}\;E_{f\;,\;g}{\left[\delta_{2}\right]}^{\rho }+E_{f\;,\;g}{\left[\beta_{3}\right]}^{\rho }\right]. \end{array}$$

We now study the terms on the RHS of (125) separately, starting with the fourth (last). By (85)–(90), which hold also if ρ>1,

(126) $$\begin{array}{c} \sum_{x,y}P\left(x,y\right)\;E_{f\;,\;g}{\left[\beta_{3}\right]}^{\rho }\leq 2^{-\rho \epsilon }. \end{array}$$

As for the first term on the RHS of (125),

(127) $$\begin{array}{c} \sum_{x,y}P\left(x,y\right)\;E_{f\;,\;g}\left[\beta_{3}\right]\leq 2^{-\epsilon }, \end{array}$$

which follows from (126) in the same way as (97) followed from (95). As for the second term on the RHS of (125),

$$\begin{array}{cc} \sum_{x,y}P & \left(x,y\right)\sum_{y^{\prime }\neq y}\frac{1}{\left|V\right|}\;E_{f\;,\;g}{\left[\delta_{1}\right]}^{\rho } \end{array}$$

(128) $$\begin{array}{c} =\sum_{x,y}P\left(x,y\right)\sum_{y^{\prime }\neq y}\frac{1}{\left|V\right|}{\left[\;\sum_{x^{\prime }\neq x}\psi \left(x^{\prime },y^{\prime }\right)\frac{1}{\left|U\right|}\right]}^{\rho } \end{array}$$

(129) $$\begin{array}{cc} & \leq \sum_{x,y}P{\left(x,y\right)}^{\tilde{\rho }}\sum_{y^{\prime }}\frac{1}{\left|V\right|}{\left[\sum_{x^{\prime }}P{\left(x^{\prime },y^{\prime }\right)}^{\tilde{\rho }}\frac{1}{\left|U\right|}\right]}^{\rho } \end{array}$$

(130) $$\begin{array}{c} =\sum_{x,y}P{\left(x,y\right)}^{\tilde{\rho }}\sum_{y^{\prime }}{\left[\sum_{x^{\prime }}P{\left(x^{\prime },y^{\prime }\right)}^{\tilde{\rho }}\frac{1}{{\left|U\times V\right|}^{\rho }}\right]}^{\frac{1}{\rho }}\cdot {\left[\sum_{x^{\prime }}P{\left(x^{\prime },y^{\prime }\right)}^{\tilde{\rho }}\frac{1}{{\left|U\right|}^{\frac{\rho }{1+\rho }}}\right]}^{\left(1+\rho \right)\cdot \frac{\rho -1}{\rho }} \end{array}$$

(131) $$\begin{array}{cc} & \leq \sum_{x,y}P{\left(x,y\right)}^{\tilde{\rho }}{\left\{\sum_{y^{\prime }}\sum_{x^{\prime }}P{\left(x^{\prime },y^{\prime }\right)}^{\tilde{\rho }}\frac{1}{{\left|U\times V\right|}^{\rho }}\right\}}^{\frac{1}{\rho }}\cdot {\left\{\sum_{y^{\prime }}{\left[\sum_{x^{\prime }}P{\left(x^{\prime },y^{\prime }\right)}^{\tilde{\rho }}\frac{1}{{\left|U\right|}^{\frac{\rho }{1+\rho }}}\right]}^{1+\rho }\right\}}^{\frac{\rho -1}{\rho }} \end{array}$$

(132) $$\begin{array}{c} ={\left\{\frac{1}{{\left|U\times V\right|}^{\rho }}{\left[\sum_{x,y}P{\left(x,y\right)}^{\tilde{\rho }}\right]}^{1+\rho }\right\}}^{\frac{1}{\rho }}\cdot {\left\{\frac{1}{{\left|U\right|}^{\rho }}\sum_{y^{\prime }}{\left[\sum_{x^{\prime }}P{\left(x^{\prime },y^{\prime }\right)}^{\tilde{\rho }}\right]}^{1+\rho }\right\}}^{\frac{\rho -1}{\rho }} \end{array}$$

(133) $$\begin{array}{cc} & \leq {\left(2^{-\rho \epsilon }\right)}^{\frac{1}{\rho }}\cdot {\left(2^{-\rho \epsilon }\right)}^{\frac{\rho -1}{\rho }} \end{array}$$

(134) $$\begin{array}{cc} & =2^{-\rho \epsilon }, \end{array}$$

where in (129), we extended the inner summations and used that $\psi \left(x^{\prime },y^{\prime }\right)\leq {\left[P\left(x^{\prime },y^{\prime }\right)/P\left(x,y\right)\right]}^{\tilde{\rho }}$; (131) follows from Hölder’s inequality; and (133) follows from (89)–(90) and (81)–(82). In the same way, we obtain for the third term on the RHS of (125):

(135) $$\begin{array}{c} \sum_{x,y}P\left(x,y\right)\sum_{x^{\prime }\neq x}\frac{1}{\left|U\right|}\;E_{f\;,\;g}{\left[\delta_{2}\right]}^{\rho }\leq 2^{-\rho \epsilon }. \end{array}$$

From (125), (127), (134), (135), and (126), we deduce:

(136) $$\begin{array}{cc} \sum_{x,y}P\left(x,y\right)\;E_{f\;,\;g}\left[\beta_{3}^{\rho }\right] & \leq 2\cdot 4^{\rho^{2}}\left(2^{-\epsilon }+2^{-\rho \epsilon }+2^{-\rho \epsilon }+2^{-\rho \epsilon }\right) \end{array}$$

(137) $$\begin{array}{c} \leq 8\cdot 4^{\rho^{2}}\cdot 2^{-\epsilon }, \end{array}$$

where (137) holds because $2^{-\rho \epsilon }\leq 2^{-\epsilon }$ since ρ>1 and ϵ>0. Finally, (68), (99), (100), and (137) imply:

(138) $$\begin{array}{cc} E_{f\;,\;g}\left[E[G{\left(X,Y|f\left(X\right),g\left(Y\right)\right)}^{\rho }]\right] & \leq 1+4^{\rho }\left(2\cdot 2^{\rho^{2}+1}\cdot 2^{-\epsilon }+8\cdot 4^{\rho^{2}}\cdot 2^{-\epsilon }\right) \end{array}$$

(139) $$\begin{array}{c} \leq 1+4^{{\left(\rho +1\right)}^{2}}\cdot 2^{-\epsilon } \end{array}$$

and thus prove the existence of f:X→U and g:Y→V satisfying (64). ☐

**Corollary** **2.** *For any ρ>0, rate pairs in the interior of R(ρ) are achievable.*

**Proof.**  Let $\left(R_{X},R_{Y}\right)$ be in the interior of R(ρ). Then, (6)–(8) hold with strict inequalities, and there exists a δ>0 such that for all sufficiently large *n*,

(140) $$\begin{array}{cc} \log\lfloor 2^{nR_{X}}\rfloor & \geq H_{\tilde{\rho }}\left(X^{n}|Y^{n}\right)+n\delta , \end{array}$$

(141) $$\begin{array}{cc} \log\lfloor 2^{nR_{Y}}\rfloor & \geq H_{\tilde{\rho }}\left(Y^{n}|X^{n}\right)+n\delta , \end{array}$$

(142) $$\begin{array}{cc} \log\left\lfloor 2^{nR_{X}}\right\rfloor +\log\left\lfloor 2^{nR_{Y}}\right\rfloor & \geq H_{\tilde{\rho }}\left(X^{n},Y^{n}\right)+n\delta . \end{array}$$

Using Theorem 3 with $X^{\prime }≜X^{n}$, $Y^{\prime }≜Y^{n}$, $U≜\{1,\ldots ,\left\lfloor 2^{nR_{X}}\right\rfloor \}$, $V≜\{1,\ldots ,\left\lfloor 2^{nR_{Y}}\right\rfloor \}$, $P_{X^{\prime }Y^{\prime }}≜P_{X^{n}Y^{n}}$, and $\epsilon_{n}≜n\delta$ shows that, for all sufficiently large *n*, there exist encoders $f_{n}:X^{n}\to U$ and $g_{n}:Y^{n}\to V$ and a guessing function $G_{n}$ satisfying:

(143) $$\begin{array}{cc} E\left[G_{n}{\left(X^{n},Y^{n}|f_{n}\left(X^{n}\right),g_{n}\left(Y^{n}\right)\right)}^{\rho }\right] & \leq \left\{\begin{array}{cc} 1+4^{\rho +1}\cdot 2^{-\rho \epsilon_{n}} & \mathrm{if}\;\rho \in (0,1],\\ 1+4^{{\left(\rho +1\right)}^{2}}\cdot 2^{-\epsilon_{n}} & \mathrm{if}\;\rho >1. \end{array}\right. \end{array}$$

Because $\epsilon_{n}$ tends to infinity as *n* tends to infinity, the RHS of (143) tends to one as *n* tends to infinity, which implies that the rate pair $\left(R_{X},R_{Y}\right)$ is achievable.  ☐
